# A New Alternative Nutritional Source Hawthorn Vinegar: How It Interacts with Protein, Glucose and GLP-1

**DOI:** 10.3390/nu16132163

**Published:** 2024-07-08

**Authors:** Nilay Seyidoglu, Deniz Karakçı, Hale Ergin Eğritağ, Seydi Yıkmış

**Affiliations:** 1 Department of Physiology, Faculty of Veterinary Medicine, Tekirdag Namik Kemal University, Tekirdag 59030, Türkiye; 2 Department of Biochemistry, Faculty of Veterinary Medicine, Tekirdag Namik Kemal University, Tekirdag 59030, Türkiye; dkarakci@nku.edu.tr; 3 Department of Biochemistry, Faculty of Veterinary Medicine, Burdur Mehmet Akif University, Burdur 15030, Türkiye; vhhaleergin@gmail.com; 4 Department of Food Technology, Tekirdag Namik Kemal University, Tekirdag 59030, Türkiye

**Keywords:** glucagon-like peptide-1 (GLP-1), glucose, hawthorn vinegar, protein, rat, ultrasound-treated

## Abstract

(1) Background: There is a balance between nutrition, glycemic control, and immune response. Their roles in physiological mechanisms are essential for maintaining life quality. This study aimed to evaluate hawthorn vinegar’s metabolic effects, and describe its possible mechanism. We also pointed out several vinegar production methods to clarify the antioxidant features. (2) Methods: In the study, three vinegar techniques were applied to vinegar: traditional production of hawthorn vinegar (N), thermal pasteurization (P), and ultrasound method (U). Thirty-two female adult Wistar albino rats were randomly separated into four groups: Control, N1 (regular vinegar; 1 mL/kg bw), P1 (pasteurized vinegar; 1 mL/kg bw), and U1(ultrasound treated vinegar; 1 mL/kg bw). Vinegar was administered by oral gavage daily for 45 days. Initial and final weights, the percentage changes of body weight gains, and Gamma-Glutamyl Transferase (GGT) values of plasma and liver were measured. The total protein, globulin, and albumin values of plasma, liver, and intestinal tissue were determined. In addition, plasma glucagon-like peptide-1 (GLP-1) and glucose concentrations were evaluated. (3) Results: There was a statistical increase in total intestinal protein value and an increasing tendency in total protein in plasma and liver in group U1 compared to group Control. However, the GGT concentrations in plasma and liver were slightly lower in group U1 than in group Control. In addition, there were significant increases in plasma GLP-1 values in all experimental groups compared to the Control group (*p*: 0.015; 576.80 ± 56.06, 773.10 ± 28.92, 700.70 ± 17.05 and 735.00 ± 40.70; respectively groups control, N1, P1, and U1). Also, liver GLP-1 concentrations in groups P1 and U1 were higher than in group Control (*p*: 0.005; 968.00 ± 25.54, 1176 ± 17.54 and 1174.00 ± 44.06, respectively groups control, P1 and U1). On the other hand, significant decreases were found in plasma glucose concentrations in groups N1 and U1 as to the Control group (*p*: 0.02; Control: 189.90 ± 15.22, N1: 133.10 ± 7.32 and U1: 142.30 ± 4.14). Besides, liver glucose levels were lower in all experimental groups than in group Control statistically (*p*: 0.010; 53.47 ± 0.97, 37.99 ± 1.46, 44.52 ± 4.05 and 44.57 ± 2.39, respectively groups control, N1, P1, and U1). (4) Conclusions: The findings suggest that hawthorn vinegar can balance normal physiological conditions via intestinal health, protein profiles, and glycemic control. Additionally, ultrasound application of vinegar may improve the ability of hawthorn vinegar, and have positive effects on general health.

## 1. Introduction

Hawthorn is a wild fruit that has been known since ancient times. Modern research shows its beneficial properties and therapeutic effects on health. Its genus, *Crataegus*, belongs to the Rosaceae family with several hundred species of shrubs and trees. Also, it can be cultivated in several regions around the world. The most common hawthorn types are found in Europe, Asia, Africa, and North America. In Türkiye, *Crataegus tanacetifolia* is the endemic member of the fruit [1]. The plant has thorns and dark red fruits. The leaves, seed traits, and fruit color are highly diverse. There are approximately 1–5 fruit leaves that simple, row coils, lobed, or virtually. The hawthorn fruit is similar to a berry, and also anatomically likes a stone [2]. Researchers demonstrated that hawthorn contains valuable biological compounds, including tannin, saponin, vitamins, fatty acids, and trace elements. In addition, it was reported that its leaves, flowers, and fruits include sugars, sugar alcohols, glucose, and essential oils [3]. Nevertheless, researchers have also determined several antioxidant compounds such as epicatechin, flavonoids, phenolic compounds, polyphenols, hyperoside, and phytosterols [3,4]. Besides that, the fruit includes lignin, ascorbic acid, and other essential acids such as tartaric, salicylic, and quinic acid, which have roles in anti-inflammatory action [5,6]. In this context, hawthorn has been drawn attention as an ideal antioxidant and anti-inflammatory agent due to its rich bioactive contents [6].

People have utilized hawthorn and its extracts as an herbal remedy for centuries to protect their health and prevent diseases. Moreover, several studies have mainly focused on the biological features and health benefits of hawthorn fruit and its extracts [7]. Kwok et al. [8] showed that ethanolic extract of hawthorn can affect the reactive oxygen species scavenging, and the cholesterol value decreases in rats due to its phenolic compounds. The flavonoids in hawthorn can modulate the pro-inflammatory and anti-inflammatory cytokines, and alleviate the inflammation of intestinal tissue [9]. Martínez-Rodríguez et al. [10] reported that hawthorn has significant hepatoprotective effects in rats, especially reducing cholesterol, triglycerides and liver enzyme values. In addition, researchers indicated that hawthorn has positive actions on the regulation of glycogen, antioxidant activity, and, thereby health [11,12]. According to its nutritional value, some traditional products of hawthorn, such as bakery, canned, chips, and vinegar, are consumed by people as well as used as a medical remedy.

The normal physiological conditions of a mammalian process begin with protecting intestinal health and blood glycemic control. Glucose is required for normal body function due to its regulation with insulin. The modulation of circulatory proteins, and the balance between glucose and glucagon-like peptide-1 (GLP-1) secretion lead to a physiological response in normal physiological process. GLP-1 is an essential endocrine hormone that stimulates the glucose-dependent insulin secretion from pancreatic cells. It has also been speculated that GLP-1 can improve the pancreatic beta cells, and normalizes the blood glucose, and thereby restores the insulin secretion [13]. Studies showed that hawthorn polyphenol and flavonoid extracts can reduce blood glucose, and regulates homeostasis [14,15,16,17]. Aierken et al. [14] found that hawthorn extracts can decrease blood glucose, and increase plasma insulin releasing in diabetes. Wang et al. [17] reported that increase of postprandial glucose is inhibited by flavonoids of hawthorn in rats. They were determined that hawthorn can protect the heart against cardiomyopathy in diabetic situations. In addition, Xin et al. [16] studied quercetin and hyperoside in polyphenols in hawthorn berries, and they showed the inhibition of alpha-glucosidase in diabetic rats. This biological activity is essential for reducing blood glucose and regulating homeostasis by hawthorn [16].

The role of nutrition in metabolism with natural foods, supplements, extracts, and vinegar has been interested in researchers over the past few years due to pandemic diseases, influenza, and important metabolic diseases [18,19]. Among these nutrient sources, vinegar is a popular food staple utilized as a digestive aid, and a drug for medical conditions. There are many types of vinegar produced from fruits and plants. The common vinegars are made from raw plant materials such as apples, grapes, sugarcanes, and grains [20]. Depending on the type, vinegars are excellent sources of phytochemicals, minerals, proteins, and vitamins. Vinegars contain plant chemicals and polyphenols, which are also known as antioxidants. It was reported that due to their antioxidant and biological features, vinegars can protect cells from oxidative stress, and thereby prevent metabolism [21]. Several studies observed that daily vinegar intake could improve the lifestyle-related diseases, health and life quality [22]. According to the literatures, hawthorn vinegar positively affects some diseases, especially hyperglycemia, dyslipidemia, atherosclerosis, and blood pressure [23,24,25]. Nevertheless, vinegars have different fermentation procedures and raw materials that give unique tastes, odors, and flavors. Some production methods, especially ultrasound-treated processes, improve the bioactive features of vinegar [26]. This method is a non-thermal technology that activates the antioxidant features, inactivates the microbial status, impacts the quality parameters, and heals the functions of liquid foods. In addition, the ultrasound method can improve the antioxidant efficiency of vinegar.

Based on the literature, polyphenols, and flavonoid compounds of hawthorn have a positive ability on protein and glucose metabolism. The researchers observed that proteins and antioxidants have crucial roles in biological reactions. Insight of health management, nutrient sources have been critical to protect and support the health and life quality in last years. According to scientific opinions, people have shown an increasing tendency to use various nutrient sources in last years, especially vinegar. In this context, the current study was carried out to investigate the effects of hawthorn vinegar, produced in three different methods, on plasma proteins, glucose and GLP-1 parameters in adult rats. Also, we addressed the effects of hawthorn vinegar produced by ultrasound method, and its effectiveness in improving physiological status and health.

## 2. Materials and Methods

### 2.1. Experimental Animal

In this study, thirty-two female Wistar albino rats weighing 230–270 g and aged 6–7 months were used for the experiment. The rats were housed in plastic and clear cages under standard laboratory conditions (22 ± 1.0 °C 55 ± 10% humidity). Stainless steel food hoppers and wood shavings for bedding material were used.

The rats were given ad libitum access to a commercial pelleted rodent diet and tap water. The basal diet contained 2000–2500 kcal/kg of energy, and the content of raw protein (23%), crude oil (3%), crude cellulose (7%), and ash (8%), which was prepared by a commercial company [27]. Before the experiment, rats were given one week to acclimate to laboratory conditions.

Hawthorn vinegar was given to the rats by oral gavage daily within a high dose for each group throughout the 45 trial days. Groups were designed as follows;

1. Group: Control (basal diet)
2. Group: N1 (1 mL/kg Untreated Hawthorn Vinegar daily)
3. Group: P1 (1 mL/kg Thermal Pasteurized Hawthorn Vinegar daily)
4. Group: U1 (1 mL/kg Hawthorn Vinegar Ultrasound treated daily)

### 2.2. Hawthorn Vinegar

The hawthorn fruit (*Crataegus tanacetifolia*) samples were collected from a commercial company in Bursa/Turkey, and then the untreated hawthorn vinegar was produced using the conventional vinegar manufacturing method [28]. The vinegars were processed by 3 methods that modified the obtained vinegar by Yıkmış [26]. As a result of fermentation, hawthorn vinegar showed an acetic acid content of approximately 4%. The first method is the vinegar without any treatment (Group N1). Second is the thermal pasteurization obtained from the vinegar (Groups P1). The third method is the response surface method and ultrasound-treated vinegar (Groups U1). The processes of untreated hawthorn vinegar and vinegar methods, and the determination of bioactive compounds of vinegar, were explained in our previous research [25].

### 2.3. Measurements

The body weight parameters are important indicators of animal distress or discomfort. The body weights of rats were measured at the beginning and the end of the trial, and the percentage changes of body weight gains were calculated to identify the animals’ normal health condition. At the end of the study, the blood samples were obtained by puncturing the heart under isoflurane anesthesia. The blood samples were centrifuged at 3000 rpm for 10 min on the same day to separate the plasma.

The liver and intestinal tissue samples were separated into pieces of appropriate size for homogenization (1 g tissue). The samples were placed in PBS (pH 7.4) and adjusted to tissue weight (1 g tissue: 9 mL PBS). The samples were homogenized with a homogenizer (Interlab, Istanbul, Turkey) by centrifuged at 5000× *g* for 5 min. Then, the supernatants were transferred into microtubes and stored at −80 °C until the analysis day.

Plasma, liver and intestinal tissues of Total Protein, Albumin, Globulin, Gamma-Glutamyl Transferase (GGT), and Glucose (Biolabo A.S.S., Maizy, France) concentrations were analyzed by the spectrophotometric method. Also, Glucagon-like Peptide-1 (GLP-1) concentrations (Rat-Glucagon-like Peptide-1, Bt Lab, Shanghai, China, Cat No: E0918 Ra) in plasma and liver tissues were defined by the ELISA method. All of the biochemical parameters were performed with a microplate reader (Epoch, BioTek, Winooski, VT, USA).

Spectrophotometric Method: Reagents and specimens were kept at room temperature. For each tube, 1000 μL reagent was added. Then, blank, standard, and samples were loaded with 10 μL into the reagent. They were mixed gently and let them stand for 5 min. at 37 °C in the incubator. Absorbances were recorded at 500 nm in the spectrophotometer. For the calculated data, sample absorbances were divided into standard absorbances and then multiplied by the standard concentration.

ELISA Method: The Elisa plate included in the kit had been pre-coated with a specific antibody. Standards or samples were loaded into the appropriate plate wells and mixed with the specified antibody. Then, each plate well was treated with a particular antibody conjugated with Horseradish Peroxidase (HRP) and incubated for 1 h at 37 °C. The free components were rinsed away five times. Each well was treated with TMB substrate solution. Only the wells containing HRP-conjugated antibodies appeared blue before becoming yellow after adding the stop solution. The optical density (OD) was measured spectrophotometrically at a wavelength of 450 nm. The OD value was proportional to the concentration of the GLP-1.

### 2.4. Statistical Analyses

Statistical analysis was performed with Graph Pad Prism Graphical–Statistical package version 5 (30 days demo version). Data were expressed as mean ± SE. For comparison between groups (more than two), ANOVA was used, followed by Tukey test as post hoc. For interpretation of results, α = 0.05 was used for level of significance.

## 3. Results

In the study, the total phenolic and flavonoid contents of all groups were measured to assess the antioxidant activity of the ultrasound method according to our previous study [25]. The total phenolic (116.99 mgGAE/100 mL), total flavonoid (15.89 mgCE/100 mL), DPPH (62.35%), and CUPRAC (67.39%) values of hawthorn vinegar were the highest concentrations in ultrasound method in group U1. Nevertheless, antioxidant concentrations in thermal pasteurization processing method (total phenolic: 104.22 mgGAE/100 mL, total flavonoid: 13.18 mg CE/100 mL, DPHH:54.86% and CUPRAC: 60.22%) were lower than standard vinegar method (total phenolic: 110.58 mgGAE/100 mL, total flavonoid: 14.22 mg CE/100 mL, DPHH:57.39% and CUPRAC:63.55%).

**Figure 1:**
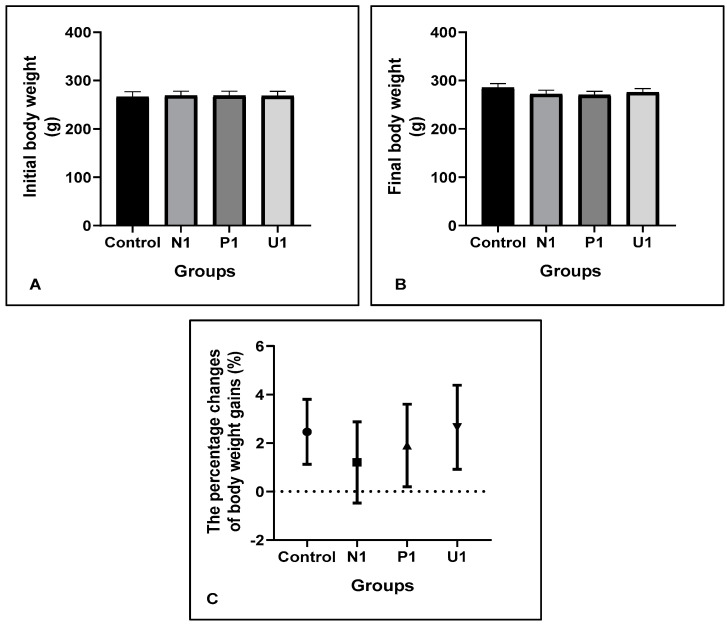
Effects of hawthorn vinegar on body weight parameters in all groups. (**A**) Initial body weight. (**B**) Final body weight. (**C**) The percentage changes of body weight gains. All data are presented as the mean ± SE (n = 8).

The body weights and body weight gain changes are important indicators for measuring animals’ distress, pain or discomfort in a trial. Our results showed that there were no differences in initial (*p* < 0.05; 266.20 ± 10.76, 269.10 ± 8.88, 268.70 ± 9.43 and 268.30 ± 9.54, respectively, groups Control, N1, P1 and U1) and final body weights (*p* < 0.05; 285.10 ± 8.70, 272.00 ± 7.95, 270.00 ± 7.70 and 275.70 ± 7.51, respectively, groups Control, N1, P1 and U1), and also percentage changes of body weight gains (*p* < 0.05; 2.46 ± 1.33, 1.20 ± 1.67, 1.90 ± 1.70 and 2.65 ± 1.73, respectively, groups Control, N1, P1 and U1) among all groups, which were recorded weekly for measuring distress or discomfort of animals (Figure 1).

The concentrations of total protein, globulin, albumin, and globulin/albumin ratio in plasma, liver, and intestinal tissues are shown in Table 1. Plasma total protein value slightly increased in the U1 group compared to the Control group (7.07 ± 0.41 and 7.16 ± 0.50, Control and U1 groups, respectively). In addition, there was an increasing tendency in protein levels of liver tissues in group U1 as to group Control (4.11 ± 0.19 and 4.40 ± 0.20 respectively, Control and U1). However, there is a statistical increase in protein value in intestinal tissue in group U1 than the Control group (*p*: 0.015; 3.22 ± 0.08 and 3.77 ± 0.24, Control and U1 groups respectively).

**Figure 2:**
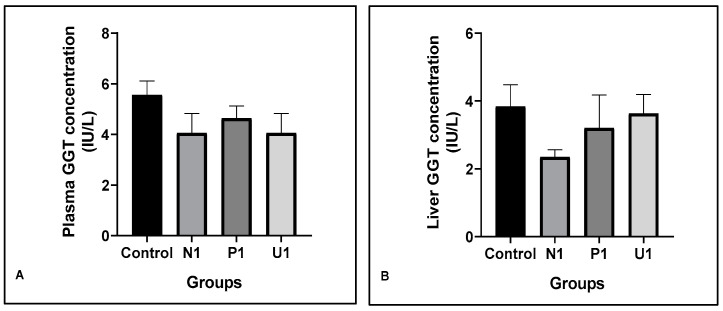
Effects of hawthorn vinegar for 45 days on plasma and liver tissue GGT concentrations in all groups. (**A**) Plasma GGT con. (**B**) Liver tissue GGT con. All data are presented as the mean ± SE (n = 8).

The liver Gamma-Glutamyl Transferase enzyme (GGT) is accepted as a diagnostic marker for health or stress conditions. In the present study, the plasma and liver GGT values are presented in Figure 2. The plasma and liver GGT values were similar in all experimental groups compared to the Control group. Although not significant, plasma and liver GGT values decreased in group U1 as compared to group Control (Plasma GGT: 5.55 ± 0.56 and 4.05 ± 0.77; liver GGT: 3.84 ± 0.64 and 3.63 ± 0.56, respectively, groups control and U1).

**Figure 3:**
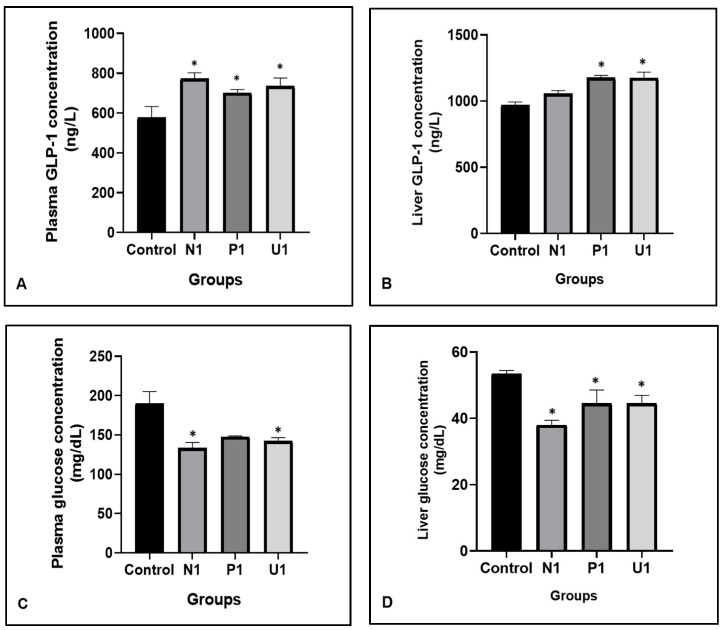
Effects of hawthorn vinegar for 45 days on plasma and liver tissue GLP-1 and glucose concentrations in all groups. (**A**) Plasma GLP-1 con. (**B**) Liver tissue GLP-1 con. (**C**) Plasma glucose con. (**D**) Liver glucose con. All data are presented as the mean ± SE (n = 8). * *p* < 0.05 when compared with control rat values.

Glucagon-like peptide-1 (GLP-1) is a physiological regulator hormone of metabolic control. The peptide is accepted to normalize the blood glucose level. In the present study, GLP-1 and glucose values are stated in Figure 3. The plasma GLP-1 values were found to be high in all experimental vinegar groups compared to the Control group (*p*: 0.015; 576.80 ± 56.06, 773.10 ± 28.92, 700.70 ± 17.05 and 735.00 ± 40.70; respectively groups Control, N1, P1 and U1). There were statistical increases in liver GLP-1 levels in groups P1 and U1 than in group Control (*p*: 0.005; 968.00 ± 25.54, 1176 ± 17.54 and 1174.00 ± 44.06, respectively groups control, P1 and U1). Besides that, plasma glucose levels had a decrease in groups N1 and U1 compared to the Control group (*p*: 0.02; Control: 189.90 ± 15.22, N1: 133.10 ± 7.32 and U1: 142.30 ± 4.14). Also, liver glucose values were decreased in groups N1, P1 and U1 compared to group Control statistically (*p*: 0.010; 53.47 ± 0.97, 37.99 ± 1.46, 44.52 ± 4.05 and 44.57 ± 2.39, respectively groups Control, N1, P1 and U1).

## 4. Discussion

There is still a mystery lies between food and medicine. For millennia, some traditional herbs and plants have been used as ready-to-use healing foods, and also as therapeutic remedies. The World Health Organization (WHO) and Food and Drug Administration (FDA) are concerned mainly with safety issues and interactions with conventional medicines. Todays, people use various medicinal herbs and their extracts. Among these nutrients, hawthorn is an exciting nutrient source. The current screening, as well as previous studies, has illustrated that hawthorn and its extracts improve the blood protein, glucose, and homeostasis. However, there have been still limited studies into hawthorn vinegar and its efficiencies. The recent study provides the positive actions of hawthorn vinegar produced by ultrasound processing and thermal pasteurization on protein and glucose metabolism. Also, improving the protein status and antioxidative metabolism features with vinegar produced by ultrasound method are underlined in the findings.

The body weight parameters and serum enzyme activities are the most important ways to diagnose physiological conditions of animals. The body weights and body weight gains are important features to identify the distress and discomfort conditions of animals. Particularly, body weight loss is considered as a sign of pain and discomfort [29]. In our study, initial and final body weights, and the percentage changes of body weight gains in all experimental groups were similar to group Control. The results showed that rats adapted equally well to day meals and vinegar in the study days. Additionally, the Gamma-Glutamyl Transferase enzyme (GGT) is an essential biomarker of cellular antioxidant inadequacy, and also of the risk of diseases. Some researchers reported that increased plasma or liver GGT can be a marker for oxidative stress [30,31]. Similar to our results, the plasma and liver GGT were found similar in all groups, especially in ultrasound treated vinegar group (Plasma GGT: 5.55 ± 0.56 and 4.05 ± 0.77; Liver GGT: 4.03 ± 0.41 and 3.63 ± 0.56, respectively groups Control and U1). So, it can be suggested that the 1 mL/kg bw dose of hawthorn vinegar can be acceptable for nutrition and general health.

Based on the literature, plasma protein, and globulin are important molecules for normal physiological and immunological status. The researchers observed that proteins have crucial roles in biological reactions. Initially, it’s accepted that proteins can act on buffer systems, and can control blood circulation, and thereby altering the immunological status and then maintaining homeostasis [32,33,34]. Normally, plasma protein is a form of reserve protein. It’s also secreted into intestinal tissue, then digested and reabsorbed as amino acids. The high total plasma protein observes an improved ability of the hepatocytes to synthesize protein. Therefore, high protein enhances the physiological and immunological situation [35]. In our study, protein levels of plasma (7.07 ± 0.41 and 7.16 ± 0.50, respectively, Control and U1 groups) and liver (4.11 ± 0.19 and 4.40 ± 0.20, respectively, Control and U1 groups) tended to increase in group U1 compared to group Control. However, intestinal tissue protein was higher in group U1 than in group Control significantly (*p*: 0.015; 3.22 ± 0.08 and 3.77 ± 0.24, respectively, control and U1 groups). Besides high total protein, the globulin concentrations of plasma, liver, and intestinal tissues in rats fed 1 mL/kg bw hawthorn vinegar (U1) were higher than the Control group, although not significant. Similar to our findings, Tlatelpa-Morales et al. [36] found that acetone extract of hawthorn protects the blood proteins. Ahmadipour et al. [34] studied hawthorn extract in drinking water, and they determined higher serum protein concentrations in broilers. Also, Khalil et al. [37] reported the increased total protein in rats when feeding alcoholic extract of hawthorn. They indicated that there is an affinity between hawthorn extract and plasma protein. These results potentially mean better intestinal health and immune status. In addition, because of globulin level and its correlation with immunoglobulins, a high globulin level has been suggested an enhancer for immune status [32,34]. Moreover, researchers reported that hawthorn polyphenols and flavonoids compounds have a positive ability on protein metabolism and glucose [38]. Thereby, it can be said that 1 mL/kg bw hawthorn vinegar can be an essential dose for protecting the animals’ normal healthy condition and immune status.

Dietary proteins and peptides can improve plasma glucagon-like peptide-1 (GLP-1) secretions, thereby can control the glycemic mechanism. GLP-1 is a molecule that stimulates the secretion of insulin, and maintains the glucose level in the blood [39]. More specifically, GLP-1 can reduce the blood glucose after feeding [39]. It has been characterized as a regulator of appetite and food intake that directly induces an anorectic pathway [18,40]. Many nutrients, such as proteins, carbohydrates, and lipids, can stimulate GLP-1 secretion. The consensus is that carbohydrates and lipids are better stimulators for GLP-1 release than protein [18,40]. In our study, the highest plasma GLP-1 values were found in group U1 significantly (*p*: 0.015; 576.80 ± 56.06, 773.10 ± 28.92, 700.70 ± 17.05 and 735.00 ± 40.70; respectively groups Control, N1, P1 and U1). Also, statistical increases were determined in liver GLP-1 values in groups P1 and U1 than in group Control (*p*: 0.005; 968.00 ± 25.54, 1176 ± 17.54 and 1174.00 ± 44.06, respectively groups Control, P1 and U1). Hawthorn vinegar produced by ultrasound method (group U1) may be an important source for regulation of GLP-1 and glucose secretions. The findings tend to explain the biological action of hawthorn vinegar at concentration 1 mL/kg as an alternative nutrient in management of health and life quality. However, there is a lack of research on the regulation of hawthorn, its extract, and vinegar on GLP-1 and glucose mechanisms. Further studies are necessary to clarify this involvement.

Nevertheless, the plasma glucose concentration decreased statistically in groups N1 and U1 compared to group Control (*p*: 0.02; Control: 189.90 ± 15.22, N1: 133.10 ± 7.32 and U1: 142.30 ± 4.14). Also, the liver glucose values decreased in all experimental groups compared to Control significantly (*p*: 0.010; 53.47 ± 0.97, 37.99 ± 1.46, 44.52 ± 4.05 and 44.57 ± 2.39, respectively groups Control, N1, P1 and U1). Similar to our results, Aierken et al. [14] reported that hawthorn extract can reduce the plasma glucose concentration due to its inhibition role on enzyme α-glucosidase that reduces the glucose release. Also, Xin et al. [16] determined that polyphenols in hawthorn, especially quercetin and hyperoside have inhibition activities on enzyme α-glucosidase, and thereby glucose concentration can be decreased. In addition, they found that hawthorn increases insulin sensitivity that linked with the phosphorylation of AMP-activated protein kinase [16]. Furthermore, researchers suggest that a high dose of hawthorn can improve insulin resistance in hyperglycemia due to its impact on fatty acid molecules, which regulate insulin resistance in organisms.

Several types of vinegar are developed worldwide from several raw materials and different production methods. Ultrasound technology is an important new innovation for the food preservation process. Several studies with juices and vinegar treated ultrasound process have been reported by researchers [25,26,41]. Our previous study [25] indicated that the antioxidant compounds are improved by implementing ultrasound technology of hawthorn vinegar. In our study, total phenolic and flavonoid compounds were higher in ultrasound technology than in other processes. Our findings showed that because of the ultrasound process of hawthorn vinegar, antioxidant efficiencies are enhanced. Belonged this, the general physiological mechanisms can be well, and thereby health status can be improved.

## 5. Conclusions

Vinegar is considered as a nutrient source for better health and quality of life. Daily intake of a drink with vinegar can be accepted to improve the lifestyle against life-related diseases as well as to regulate the normal physiological status. The presence of proteins, antioxidants, vitamins, and other components in vinegar is responsible for its beneficial protective effect on several mechanisms. 

The results of our study indicated that vinegar obtained from hawthorn can help to normalize the protein and glycemic mechanisms. The significant changes of glucose and GLP-1 values were determined in high dose of hawthorn vinegar (1 mL/mg bw) whereas slight changes were in protein status. As a result, we suggest that 1 mL/mg vinegar produced by ultrasound method can protect and strengthen immunity and health. However, the effects of hawthorn vinegar on health conditions and life quality, as well as metabolic diseases, have not been well studied. Possible mechanisms of therapeutic functions of hawthorn vinegar are necessary to discuss as a nutrient source for health.

## Figures and Tables

**Table 1:** Effects of hawthorn vinegar for 45 days on protein profile in plasma, liver, and intestinal tissues in all groups. All data are presented as the mean ± SE (n = 8).

		Groups		
Parameters	Control	N1	P1	U1
Plasma total protein (g/dL)	7.07 ± 0.41	6.28 ± 0.36	6.68 ± 0.36	7.16 ± 0.50
Plasma albumin (g/dL)	3.59 ± 0.21	3.27 ± 0.13	3.43 ± 0.14	3.74 ± 0.16
Plasma globulin (g/dL)	3.48 ± 0.14	3.21 ± 0.19	3.38 ± 0.24	3.52 ± 0.68
Plasma albumin/globulin ratio (%)	0.99 ± 0.06	1.19 ± 0.14	1.25 ± 0.19	1.08 ± 0.16
Liver total protein (g/dL)	4.11 ± 0.19	4.56 ± 0.12	4.39 ± 0.03	4.40 ± 0.20
Liver albumin (g/dL)	2.18 ± 0.05	2.43 ± 0.12	1.93 ± 0.12	2.40 ± 0.15
Liver globulin (g/dL)	1.78 ± 0.05	2.20 ± 0.09	2.33 ± 0.13	2.36 ± 0.06
Liver albumin/globulin ratio (%)	1.11 ± 0.08	1.13 ± 0.10	0.90 ± 0.07	1.20 ± 0.03
Intestinal total protein (g/dL)	3.22 ± 0.08	2.95 ± 0.05	3.19 ± 0.06	3.77 ± 0.24 *
Intestinal albumin (g/dL)	0.66 ± 0.04	0.72 ± 0.03	0.77 ± 0.09	0.88 ± 0.19
Intestinal globulin (g/dL)	2.2 7 ± 0.21	2.28 ± 0.04	2.45 ± 0.06	2.66 ± 0.15
Intestinal albumin/globulin ratio (%)	0.28 ± 0.04	0.34 ± 0.02	0.35 ± 0.05	0.37 ± 0.10

* *p* < 0.05 when compared with control rat values.

## Data Availability

The original contributions presented in the study are included in the article, further inquiries can be directed to the corresponding author.
